# Q-Score: development of a new metric for continuous glucose monitoring that enables stratification of antihyperglycaemic therapies

**DOI:** 10.1186/s12902-015-0019-0

**Published:** 2015-05-01

**Authors:** Petra Augstein, Peter Heinke, Lutz Vogt, Roberto Vogt, Christine Rackow, Klaus-Dieter Kohnert, Eckhard Salzsieder

**Affiliations:** Institute for Diabetes “Gerhardt Katsch” Karlsburg, Greifswalder Str. 11e, 17495 Karlsburg, Germany; Diabetes Service Center Karlsburg, Greifswalder Str. 11e, 17495 Karlsburg, Germany; Ernst-Moritz-Arndt Universität Greifswald, Domstraße 11, 17487 Greifswald, Germany

**Keywords:** Diabetes mellitus, Continuous glucose monitoring, Patient-tailored therapy, Individualised diabetes management, Metric, Glycemic control

## Abstract

**Background:**

Continuous glucose monitoring (CGM) has revolutionised diabetes management. CGM enables complete visualisation of the glucose profile, and the uncovering of metabolic ‘weak points’. A standardised procedure to evaluate the complex data acquired by CGM, and to create patient-tailored recommendations has not yet been developed. We aimed to develop a new patient-tailored approach for the routine clinical evaluation of CGM profiles. We developed a metric allowing screening for profiles that require therapeutic action and a method to identify the individual CGM parameters with improvement potential.

**Methods:**

Fifteen parameters frequently used to assess CGM profiles were calculated for 1,562 historic CGM profiles from subjects with type 1 or type 2 diabetes. Factor analysis and varimax rotation was performed to identify factors that accounted for the quality of the profiles.

**Results:**

We identified five primary factors that determined CGM profiles (central tendency, hyperglycaemia, hypoglycaemia, intra- and inter-daily variations). One parameter from each factor was selected for constructing the formula for the screening metric, (the ‘Q-Score’). To derive Q-Score classifications, three diabetes specialists independently categorised 766 CGM profiles into groups of ‘very good’, ‘good’, ‘satisfactory’, ‘fair’, and ‘poor’ metabolic control. The Q-Score was then calculated for all profiles, and limits were defined based on the categorised groups (<4.0, very good; 4.0–5.9, good; 6.0–8.4, satisfactory; 8.5–11.9, fair; and ≥12.0, poor). Q-Scores increased significantly (*P* <0.01) with increasing antihyperglycaemic therapy complexity. Accordingly, the percentage of fair and poor profiles was higher in insulin-treated compared with diet-treated subjects (58.4% vs. 9.3%). In total, 90% of profiles categorised as fair or poor had at least three parameters that could potentially be optimised. The improvement potential of those parameters can be categorised as ‘low’, ‘moderate’ and ‘high’.

**Conclusions:**

The Q-Score is a new metric suitable to screen for CGM profiles that require therapeutic action. Moreover, because single components of the Q-Score formula respond to individual weak points in glycaemic control, parameters with improvement potential can be identified and used as targets for optimising patient-tailored therapies.

**Electronic supplementary material:**

The online version of this article (doi:10.1186/s12902-015-0019-0) contains supplementary material, which is available to authorized users.

## Background

Continuous glucose monitoring (CGM) is a new area in diabetes care and management [1-3]. The advantage of CGM is that daily glucose profiles can be visualised completely and precisely, allowing the identification of ‘weak points’ in glycaemic control. Each CGM record contains a wealth of data, and 48 parameters are currently available for the analysis of glucose profiles [4-10]. However, the analysis of CGM has not yet been standardised [10-12]. Studies by the Juvenile Diabetes Research Foundation Continuous Glucose Monitoring Study Group employed variables that described glucose levels (time/day above or below the target range), variability (standard deviation [SD], coefficient of variation [CV], mean amplitude of glycaemic excursions [MAGE], mean absolute rate of change [MARC]), and summary values for hypo- and hyperglycaemia (area under the curve for glucose [AUC_G_], low blood glucose index [LBGI], and high blood glucose index [HBGI]) [5,13-15]. Accordingly experts have suggested the use of parameters that allow the assessment of target range, glucose exposure, glucose variability, and hyper- and hypoglycaemia [10-12].

Mean blood glucose (MBG) is frequently used to reveal central glycaemic tendency. For evaluating glucose variability, a variety of parameters have been described, including SD, range, MAGE, the continuous overall net glycaemic action (CONGA), and the mean of daily differences (MODD) [11-13,15-20]. Hypo- and hyperglycaemic episodes are assessed based on the time spent and AUC_G_ of CGM segments that appear outside the target range, where hypoglycaemia is defined as time outside the glucose target range t_G <3.9_ and AUC_G <3.9_, and hyperglycaemia is defined as t_G >8.9_ and AUC_G >8.9_. Risk scores for hypo- or hyperglycaemia are based on the LBGI and HBGI, respectively [19,20]. The glycaemic risk assessment for diabetes equation (GRADE) was also developed for assessing glycaemic risk [16]. These parameters are valuable for clinical research. However, they are often impractical for use by the clinician in routine diabetes care.

Recent studies addressed the need for a single metric that allows for the assessment of short-term glycaemic control using CGM, similar to the way in which glycosylated haemoglobin (HbA_1c_) allows for the assessment of long-term glycaemic control [21-23]. Rawlings et al. [21] developed a graphical user interface to evaluate CGM profiles (CGM-GUIDE^©^) based on quantitative measures of glucose variability. Thomas et al. [22] described the ‘Glucose Pentagon’, which combines different summary measures derived from CGM profiles (including parameters describing glycaemic variability) and HbA1c for assessing glycaemic control. Marling et al. [23] developed a ‘consensus perceived glycaemic variability metric’ that captures the gestalt perceptions of experienced physicians using an automatic algorithm. However, none of these new methods has yet been introduced into routine diabetes care regimens.

The aim of this study was to develop a metric that facilitates objective assessments of glucose profiles and screening for profiles that require therapeutic action. Moreover, in order to allow patient-tailored therapy, we aimed to develop an automated method for identification of improvement potential in a given profile.

## Methods

### Patient data

CGM profiles and self-control data were recorded in earlier studies [24-28], which were approved by the Regional Ethics Review Board of the University of Greifswald (Germany). All included subjects provided informed consent to participate in CGM and data analysis. Data from 1,562 subjects (females/males; 499/1,063) with type 1 and type 2 diabetes (n = 48 and n = 1514, respectively) were analysed (Table 1). The mean age was 65.8 ± 9.0 years (range 39–89); duration of diabetes 10 ± 9.1 years (range 1–51); body mass index (BMI) 30.9 ± 5.4 kg/m^2^ (range 18.5–55.4). Subjects received diet-based diabetes therapy (n = 120), oral hypoglycaemic agents (OHA; n = 513), a combination of OHA and insulin (n = 439), or insulin alone (n = 490). The carbohydrate intake was 142.8 ± 37.2 (range 48–336) g carbohydrates/d. The mean HbA_1c_ was 7.0 ± 0.9% (range 4.5–13.4; 53 ± 10 mmol/mol [range 26–123]).

**Table 1:** **Patient demographics and characteristics**

		**T2DM**			**T1DM**
**Parameter**	**Diet**	**OHA**	**OHA + Insulin**	**Insulin**	**Insulin**
	**(n = 120)**	**(n = 513)**	**(n = 439)**	**(n = 442)**	**(n = 48)**
Age [years]	64.9 ± 8.2	65.7 ± 8.5	65.1 ± 8.1	68.1 ± 9.2	52.9 ± 10.4
Gender [f/m]	33 / 87	174 / 339	136 / 303	135 / 307	21 / 27
DM duration [years]	3 (2–6)	6 (3–11)	12 (8–17)	16 (9–23)	31 (21–36)
BMI [kg/m^2^]	29.3 ± 4.1	30.5 ± 5.2	32.6 ± 5.5	30.7 ± 5.2	26.0 ± 3.8
HbA1c [%]	6.3 ± 0.7	6.8 ± 0.8	7.2 ± 1.0	7.1 ± 1.0	7.6 ± 1.0

Data represent the mean ± SD or median (interquartile range) for each category.

OHA, oral hypoglycaemic agent; T1DM, type 1 diabetes mellitus; T2DM, type 2 diabetes mellitus.

### Continuous glucose monitoring

CGM profiles (72 h) were acquired with the Medtronic Gold system (Medtronic Diabetes, Northridge, CA, USA) according to the manufacturer’s instructions. CGM was performed in an outpatient setting under daily-life conditions. The quality of CGM profiles was assessed on three subsequent days, and the measures were averaged for analyses. All CGM profiles were assessed using the following parameters: MBG, median glucose level (median), SD, range, MAGE, CONGA over a 6-h period, MODD, interquartile range (IQR), t_G_ and AUC_G_ above or below the target range from 3.9 to 8.9 mmol/l, risk scores for LBGI and HBGI, and GRADE [16-20].

### Factor analysis

The factor analysis [29,30] was conducted with the FACTOR procedure available in PASW Statistics 17 (SPSS Inc., Chicago, IL, USA). Initially, all included parameters were normalised using the z-score and the correlation between all variables was determined. The number of components to be retained was first based on a scree plot. A calculation with an additional factor provided a further independent and interpretable factor.

The calculation of the Kaiser–Meyer–Olkin (KMO) measure resulted in a KMO of 0.821, which indicated that the factor model was appropriate and the sampling was highly adequate. A varimax (orthogonal) rotation was used to obtain a set of independent, interpretable factors. The resulting factor pattern was interpreted with the use of factor loadings >0.5.

### Categorisation of CGM profiles

A randomly selected subset from all CGM profiles (n = 766) was independently categorised into groups of ‘very good’, ‘good’, ‘satisfactory’, ‘fair’, and ‘poor’ metabolic control, by three diabetes specialists. The specialists had access to both the CGM profiles and the patient records that indicated the diabetes type, diabetes duration, and types of therapy associated with each CGM.

### Statistical methods

All analyses were performed with PASW Statistics 17. Results are expressed as mean ± SD or as medians and IQR. Analysis of variance was used to assess differences between groups. The strength of the dependence between two continuous variables was assessed with the Pearson’s correlation coefficient and between ordinal variables with Kendall’s tau-b correlation. The weighted Cohen’s kappa score [31] was used to assess the inter-rater reliability of the categorisation of the CGM profiles between diabetes specialists and between Q-Score and diabetes specialists. The reliability (concordance of assessments) was measured using the method proposed by Landis and Koch [32]. A *P*-value <0.05 was considered to indicate statistical significance.

## Results

### Extraction of factors accounting for the quality of CGM profiles

To identify the criteria determining a glucose profile, we performed a factor analysis. Four factors were identified (Table 2), which accounted for 95% of the common variance in the dataset (38% factor 1; 34% factor 2; 20% factor 3; and 3% factor 4). Factor 1 (central tendency and hyperglycaemia) was associated with positive loadings of MBG, median, t_G >8.9_, AUC_G >8.9_, GRADE, and HBGI. Factor 2 (within-day variability) was associated with positive loadings of range, SD, IQR, MAGE, CONGA_6-h_, and MODD. Factor 3 (hypoglycaemia) was associated with positive loadings of t_G <3.9_, AUC_G <3.9_, and LBGI. Factor 4 (between-day variability) was associated with a positive loading of MODD.

**Table 2:** **Parameters for four factors identified in the factor analysis** ^**a**^

**Parameter**	**FACTORS**			
	**1**	**2**	**3**	**4**
	**Central tendency and hyperglycaemia**	**Intra-day variability**	**Hypoglycaemia**	**Inter-day variability**
Range	0.298	**0.903**	0.156	0.077
SD	0.320	**0.920**	0.186	0.078
IQR	0.344	**0.863**	0.174	0.073
MAGE	0.259	**0.901**	0.160	0.077
CONGA-6 h	0.302	**0.916**	0.171	0.025
MBG	**0.922**	0.260	−0.264	0.055
Median	**0.931**	0.176	−0.276	0.074
t_G >8.9 mmol/l_	**0.876**	0.334	−0.139	0.101
AUC_G >8.9 mmol/l_	**0.906**	0.320	−0.027	0.032
HBGI	**0.931**	0.321	−0.070	0.044
GRADE	**0.929**	0.348	0.027	0.068
t_G <3.9 mmol/l_	−0.143	0.195	**0.951**	0.036
AUC_G <3.9 mmol/l_	−0.083	0.201	**0.954**	0.020
LBGI	−0.230	0.189	**0.942**	0.018
MODD	0.354	**0.657**	0.139	**0.649**

^a^Data are from 1,562 continuous glucose monitoring profiles.

Parameter loadings >0.5 indicate that the given factor was important for interpretation of the factor (denoted in bold).

AUC_G_, area under the curve for glucose; CONGA, continuous overall net glycaemic action; GRADE, glycaemic risk assessment for diabetes equation; HBGI, high blood glucose index; IQR, interquartile range; LBGI, low blood glucose index; MAGE, mean amplitude of glycaemic excursions; MBG, mean blood glucose; MODD, mean of daily differences; SD, standard deviation; t_G_, time outside glucose target range.

### Construction of the Q-Score

Among these factors, the parameters with loadings >0.5 were highly correlated with each other, based on linear regression functions (data not shown). Therefore, one parameter from each factor could be selected for the construction of the Q-Score. The only exception was the analysis of MBG and the time spent above the target range (t_G >8.9_) from factor 1, where a sigmoid function was found (Additional file 1: Figure S1). The time spent above the target range was highly variable for any given MBG. Therefore, these two parameters were selected from factor 1 for the construction of the Q-Score.

From all factors, we selected one parameter that had a high factor loading, was simple to calculate, and was easy to interpret in the context of a CGM curve for general practitioners. These parameters were the MBG and the time spent above the target range from factor 1; the range from factor 2; the time spent below the target range from factor 3; and the MODD from factor 4 (Additional file 1: Figure S2).

In the proposed Q-Score, all parameters are combined to generate a single measure and should, therefore, have equal weight. To achieve equivalence in the parameters for calculations, the five selected parameters with unequal means and variances were standardised with a z-transformation. The Q-Score was computed as the sum of all five standardised variables. This ensured that all five parameters had an equal impact on the Q-Score. Then, to ensure positive values, we added a constant equal to 8. The formula for the Q-Score was:

$$ Q- Score=8+\frac{MBG-7.8}{1.7}+\frac{range-7.5}{2.9}+\frac{{\mathrm{t}}_{\mathrm{G}<3.9}-0.6}{1.2}+\frac{{\mathrm{t}}_{\mathrm{G}>8.9}-6.2}{5.7}+\frac{MODD-1.8}{0.9} $$

### Categorisation of CGM profiles

**Figure 1:**
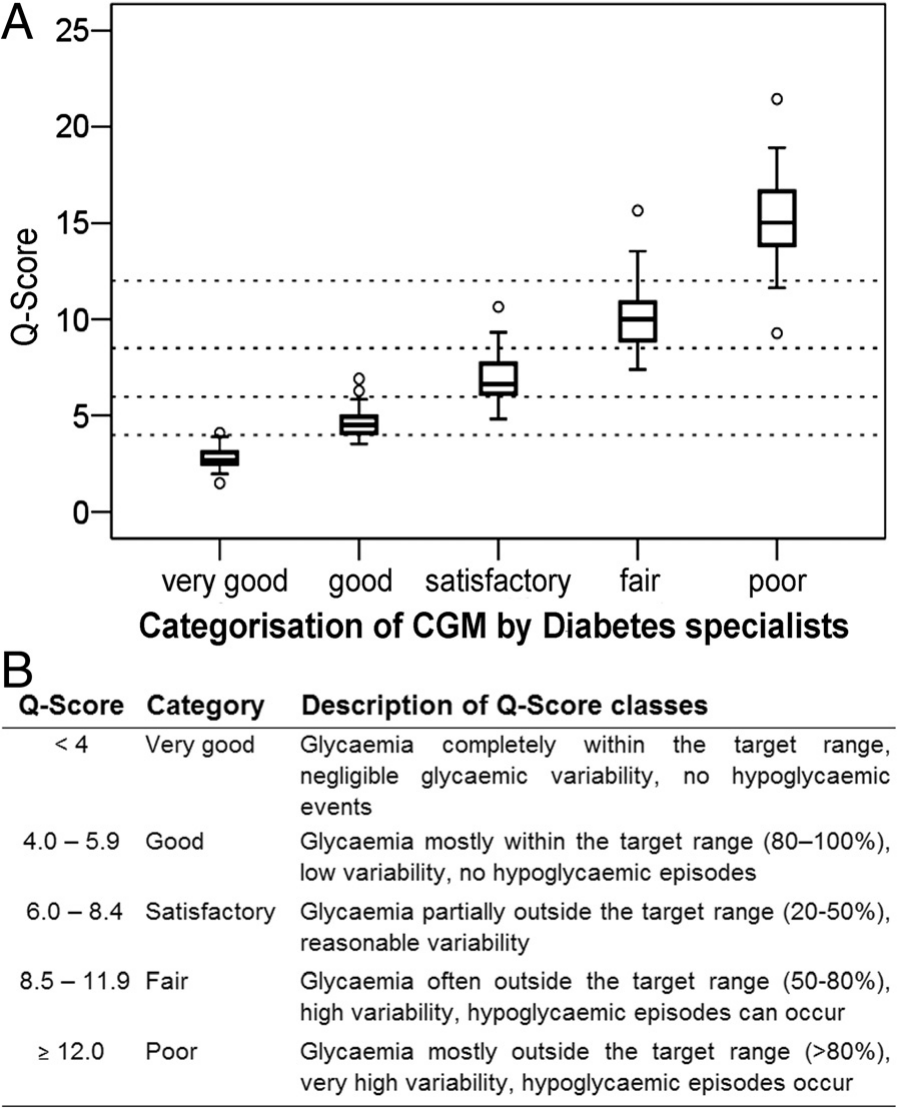
Definition of Q-Score categories. **(A)** The 766 CGM profiles were categorised by the diabetes specialist according to the metabolic control (very good, good, satisfactory, fair and poor). For each category the corresponding Q-Scores are shown as a box-plot analysis. The boundaries of the Q-Score categories are shown as dotted lines. **(B)** Description of Q-Score categories.

Three diabetes specialists evaluated independently 766 CGM profiles and categorised them based on the metabolic control revealed by the blood glucose profile as very good, good, satisfactory, fair or poor. Representative examples of CGMs with Q-Scores in different classes of metabolic control are shown in Additional file 1: Figure S3. The inter-rater reliability between the diabetes specialists using weighted Cohen’s kappa [31] was significantly different from pure chance for all diabetes specialists (0.438 ± 0.019, 0.713 ± 0.016, 0.403 ± 0.018; *P* <0.001 for all). The categorisations were highly correlated among the specialists (Kendall’s tau = 0.671, 0.787 and 0.751; *P* <0.001), allowing us to average the categories for each patient. Scores of the same 766 CGM profiles, which were categorised by the three diabetes specialists were calculated. A box-plot analysis was used to define the limiting Q-Score values for the CGM-categories defined by the diabetes specialists (Figure 1A). The Q-Scores for the CGM-categories were as follows: <4.0, very good; 4.0–5.9, good; 6.0–8.4 satisfactory; 8.5–11.9 fair; and ≥12.0 poor. These limits were also applied to define the Q-Score categories as very good, good, satisfactory, fair and poor (Additional file 1: Figure S3). The criteria for the Q-Score categories and the description of the Q-Score categories are shown in Figure 1B.

### Reliability of the Q-Score categories

The reliability of Q-Score categories was measured using the linear weighted Cohen’s kappa coefficient [31] and concordance was assessed using the scale by Landis and Koch [32]. Overall there was a substantial concordance between the assessment of CGM profiles by the diabetes specialists and the defined Q-Score categories (κ: 0.666 ± 0.010). There was substantial concordance between two diabetes specialists in terms of the Q-Score categories (Physician A κ: 0.759 ± 0.015; Physician B κ: 0.724 ± 0.015), while the third diabetes specialist showed moderate concordance (Physician C κ: 0.519 ± 0.018). Complete concordance in the selected Q-Score categories and the assessment by diabetes specialists was achieved for 59.1% of CGM profiles, a deviation of one level in the categorisation (above or below; for example diabetes specialist assessment as ‘very good’ and a Q-Score of ‘good’) in 37.4% of CGM profiles and of two levels in 3.5% of CGM profiles (above or below; for example diabetes specialist assessment as ‘very good’ and a Q-Score of ‘satisfactory’).

### Application of Q-Score in diabetes care

**Figure 2:**
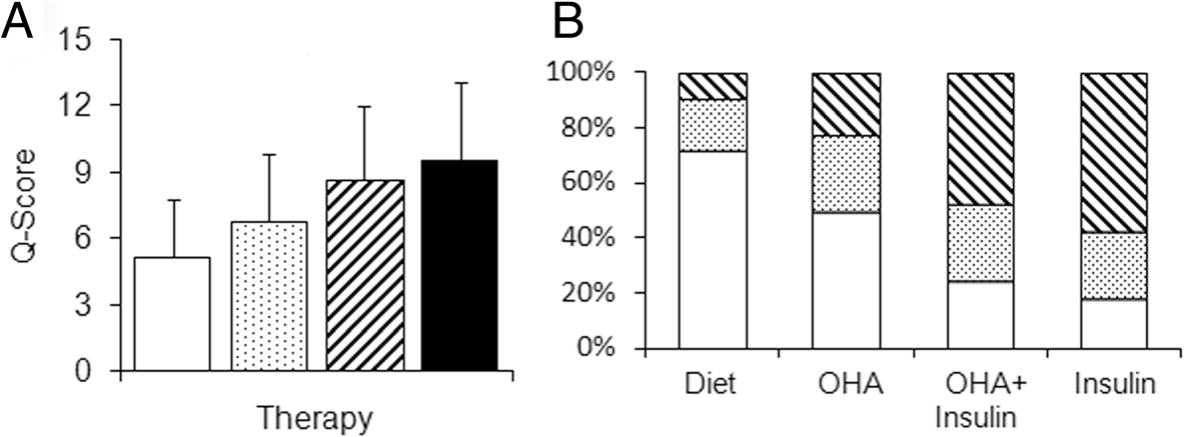
Q-Score in people with diabetes treated with diet, drugs or insulin. **(A)** Assessment of diabetes therapy using Q-Scores as indicators of short-term glycaemic control in subjects treated with diet (), oral hypoglycaemic agents (OHA; ), OHA + insulin (), and insulin alone (). Q-Scores increased significantly with the complexity of antihyperglycaemic treatments (*P* <0.001). Data represent means ± SD. **(B)** Distribution of Q-Scores, grouped as very good + good (), satisfactory (), and fair + poor () in the different therapy groups.

In the study population (n = 1,562), increases in the Q-Scores corresponded to changes in common parameters used to described glycaemic control (*P* <0.001) (Additional file 1: Table S1). We investigated whether the Q-Score also increased with the complexity of therapy (Table 3). We found that the Q-Score was lowest for subjects treated with diet (5.0 ± 2.4), increased for those treated with OHAs (6.8 ± 3.1) and OHA + insulin (8.7 ± 3.3), and was highest for subjects treated with insulin alone (9.6 ± 3.6) (Figure 2A). The analysis of the Q-Score distributions (Figure 2B) revealed significantly more fair or poor profiles in subjects treated with insulin alone compared with those treated with diet. Subjects with good and poor metabolic control were present in all treatment groups. However, the percentage of subjects with very good or good Q-Scores was decreased in insulin-treated subjects compared with those treated with diet (17.1% vs. 73.1%). Conversely, the percentage of people with fair or poor profiles was higher in insulin-treated than in diet-treated subjects (58.4 vs. 9.3%; Figure 2B). This was adequately reflected by the corresponding Q-Scores (Figure 2A, Table 3). Moreover, the Q-Score increased with rising of HbA1c. In subjects with HbA1c <6.5% (n = 531) the Q-Score was 6.23 ± 2.77 (mean ± SD). In subjects with HbA1c 6.5–6.99% (n = 375) the Q-Score was 7.56 ± 2.93 and in subjects with 7.0–7.49% (n = 322) the Q-Score was 8.62 ± 3.17. High Q-Scores (9.96 ± 3.33) were seen in subjects with HbA1c 7.5–7.99% (n = 155) and further elevated in subjects with HbA1c ≥8.0% (n = 179; 11.72 ± 3.68).

**Table 3:** **Q-Score in relation to diabetes therapy**

		**T2DM**			**T1DM**
**Parameter**	**Diet**	**OHA**	**OHA + Insulin**	**Insulin**	**Insulin**
	**(n = 120)**	**(n = 513)**	**(n = 439)**	**(n = 442)**	**(n = 48)**
Q-Score	5.1 ± 2.6	6.8 ± 3.0	8.6 ± 3.3	9.2 ± 3.4	12.7 ± 3.2
MBG (mmol/l)	6.9 ± 1.3	7.5 ± 1.6	8.0 ± 1.8	8.0 ± 1.6	8.5 ± 1.6
Range (mmol/l)	5.1 ± 2.1	6.5 ± 2.4	7.9 ± 2.7	8.5 ± 2.9	11.1 ± 2.7
t_G>8.9mmol/l_ (h)	0.9 (0.1-3.0)	3.1 (1.1-6.5)	5.5 (2.6-10.6)	6.5 (3.2-10.3)	9.1 (5.8-12.7)
t_G<3.9mmol/l_ (h)	0 (0–0.4)	0 (0–0.4)	0.1 (0–0.9)	0.2 (0–1.0)	0.9 (0.1-2.4)
MODD (mmol/l)	1.2 ± 0.5	1.5 ± 0.6	2.0 ± 0.9	2.1 ± 0.9	3.3 ± 1.1

Data represent the mean ± SD or median (interquartile range) for each category; OHA, oral hypoglycaemic agent; T1DM, type 1 diabetes mellitus; T2DM, type 2 diabetes mellitus.

### Patient-tailored analysis of CGM profiles

The Q-Score enables the identification of profiles with insufficient metabolic control. Aiming for a patient-tailored approach, we developed a method allowing the identification of the factors with improvement potential in a given glucose profile. First, we defined the limits of the improvement potential using the 95^th^ percentile of each Q-Score parameter of profiles categorised by the diabetes specialists as very good and good. Values below the 95^th^ percentile were defined as ‘appropriate’ and values above as ‘with improvement potential’. Next, the improvement potential was categorised as ‘low’, ‘moderate’ or ‘high’. Values above the 95^th^ percentile of profiles categorised as satisfactory were defined as ‘low’ improvement potential. The limits for ‘moderate’ or ‘great’ improvement potential were built, with equal class size (Additional file 1: Table S2).

**Figure 3:**
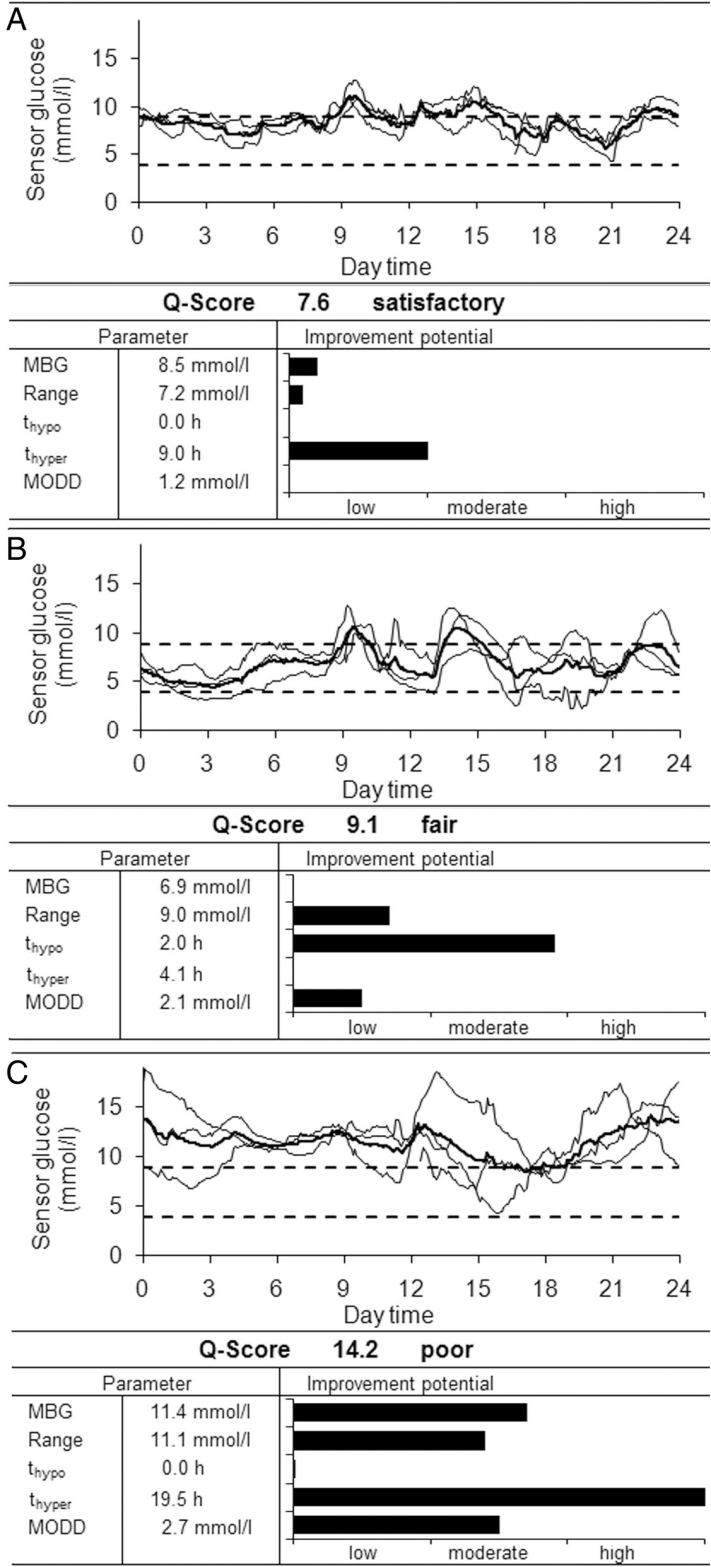
Individual improvement potential demonstrated for three CGM profiles. Values for the Q-Score parameters, and the individual improvement potential for each Q-Score parameter are demonstrated for three historic CGM curves. **(A)** The CGM profile (#128830) was recorded in a 75-year-old individual, diagnosed at age 56 (BMI 21.6 kg/m^2^, recorded HbA_1c_ 7.5%). The subject was treated with intensified conventional therapy (ICT) (prandial insulin: 5, 5, 3, 3 IU; basal insulin: 5, 6 IU). **(B)** The CGM profile (#133657) was obtained from a 53-year-old individual, diagnosed at age 46. (BMI 32.3 kg/m^2^, recorded HbA_1c_ 6.8%). The subject was treated with oral hypoglycaemic agents (metformin 1 × 850 mg, sulfonylurea 2 × 1.75 mg). **(C)** The CGM profile (#136516) was recorded in a 75-year-old individual, diagnosed at age 52 (BMI 29.7 kg/m^2^, recorded HbA_1c_ 8.6%). The subject was treated with a combination of oral hypoglycaemic agents (metformin 2 × 1000 mg) and insulin (ICT: 28, 26 and 26 IU; basal insulin 40 IU).

Overall, more than 80% of profiles categorised as fair or poor had at least three factors to optimise (Additional file 1: Figure S4). In particular, subjects with those profiles would benefit from therapy optimisation. The individual improvement potential is demonstrated by combined illustration of the CGM curve, the improvement potential for each Q-Score parameter, and the statistical data for each factor (Figure 3). Three CGM curves with different Q-Scores are provided as examples. The profile #128830 had a satisfactory Q-Score (Figure 3A). The analysis of the improvement potential indicates a low improvement potential for the time in the hyperglycaemic range. Case 133657 had a fair Q-Score (Figure 3B). The improvement potential was ‘moderate’ for t_G <3.9_ and ‘low’ for range and MODD, respectively (Figure 3B). Case 136516 had a poor Q-Score. This profile shows prolonged hyperglycaemic status, resulting in a high improvement potential for t_G >8.9_. A high MBG and increased glycaemic variability were also recorded in this subject, therefore a moderate improvement potential was recorded for central tendency, intra- and inter-daily variability (Figure 3C).

## Discussion

There is a need for a metric to assess short-term glycaemic control, similar to the way in which measuring HbA_1c_ allows the assessment of long-term glycaemic control. We aimed to develop a score-type metric that provides an overall, understandable assessment of the blood glucose profile. Ideally, this measure would be sensitive to chronic hyperglycaemia, glucose variability, and hypoglycaemia, and be applicable in routine diabetes care for the screening of profiles with insufficient metabolic control.

For the development of the metric, we first intended to identify the factors determining the quality of a CGM profile. We performed the first published factor analysis [29,30] of CGM-variables [4-7,9,10]. By definition, a factor represents a cluster of highly correlated variables [29,30]. We identified four factors that described CGM profiles: hyperglycaemia, inter- and intra-daily variability, and hypoglycaemia. To verify our findings, we analysed the variables with positive loadings within each factor. For factor 1, we found that two variables were necessary to describe hyperglycaemia: MBG, and the time spent in the hyperglycaemic range. Thus, overall, five variables adequately described the CGM: central tendency, hyperglycaemia, intra-daily variability, hypoglycaemia, and inter-daily variability. These are equivalent to the key metrics (target range, glucose exposure, glucose variability, hypoglycaemia, and hyperglycaemia) suggested by an expert panel for the standardisation of glucose reporting, analysis, and clinical decision making [10]. The new metric, which we called the Q-Score (Q = Quality), was constructed with parameters from these factors or key metrics.

Earlier studies have also sought to identify the most useful CGM-parameters for clinical use [6-8,33,34]. Rodbard [7] evaluated methods for assessment of glycaemic control and glycaemic variability. Consistent with our findings, Rodbard [7] observed high correlations among MAGE, SD, IQR, and CONGA, and also concluded that these measures provided essentially the same information. The author defined four groups of methods for characterising glucose variability. Three groups contained parameters summarised by our factor analysis in the factor ‘intra-day variability’. The fourth group [7], the MODD, was also identified in our study, belonging to factor ‘inter-day variability’. In accordance with our findings, hypoglycaemia, hyperglycaemia, and euglycaemia were identified as parameters of glycemic control [7]. Recently, Fabris et al. [35] analysed a pool of 25 glucose variability indices using the Sparse principal component analysis in a study with 17 subjects diagnosed with type 1 diabetes. The authors identified a subset of 10 different glucose variability indices that are sufficient to preserve more than the 60% of the variance originally explained by all 25 variables [35].

CGM is increasingly being introduced into diabetes care [1]; therefore, there is an increasingly urgent requirement for a metric summarising the quality of short-term glucose control [21-23]. Like us, Rawlings et al. [21] aimed to develop an integrated approach that provides a complete and consistent assessment of glycaemic control. However, these authors followed a different approach and published a graphical user interface for evaluation of CGM profiles based on glucose variability metrics [SD, MODD, CONGA(n), and MAGE] and glycaemic statistics (time spent within thresholds, time spent in hyperglycemic/hypoglycemic conditions, area under the curve, and mean glucose). The interface was tested in a small number of subjects with type 1 diabetes. Marling et al. developed a ‘consensus perceived glycaemic variability metric’ that captures the gestalt perceptions of experienced physicians using a machine learning algorithm [23]. In tests on 250 CGM profiles from subjects with type 1 diabetes, this metric outperformed mean amplitude of glycaemic excursion, standard deviation, distance travelled, and excursion frequency [23]. Thomas et al. [22] also developed a measure for assessing glycaemic control using CGM profiles. In the ‘Pentagon’ model [22] they included MBG, AUC_G >160 mg/dl_, t_G >160 mg/dl_, SD_Glucose_, and HbA_1c_; thus, that model included three of the factors identified in the present study; hyperglycaemia, central tendency and intra-daily variability. However, in contrast to the Q-Score, the Pentagon model included HbA_1c_. In another study, Thomas et al. [36] reported that the Pentagon model was helpful for assessing individual glycaemic profiles of subjects with type 1 diabetes and for assessing the influence of therapeutic interventions. In addition, they showed that model predictions of the risk of developing late complications were more accurate than HbA_1c_ predictions. Future studies are necessary to compare the Q-Score to the Pentagon model. These studies [21-23] aimed to facilitate interpretations of blood glucose profiles and to allow the identification of ‘weak points’ in diabetes management, which is consistent with our approach in this study. However, these studies focused on type 1 diabetes, whereas we tested the Q-Score in a large set of CGM profiles obtained from subjects with type 1 and type 2 diabetes. It should be noted that the relatively small number of subjects with type 1 diabetes represents a limitation of our study.

To develop a practical, readily interpreted metric for routine clinical use and screening, we intended that the Q-Score should allow categorisation of glycaemic control from very good to poor. To achieve categorisation in our study, three diabetes specialists independently classified CGM profiles into groups of very good, good, satisfactory, fair, and poor metabolic control. The majority of profiles were derived from subjects with type 2 diabetes. As expected, the results reflected subjective evaluations. However, the high Kendall’s tau correlation indicated that the results were consistent. The groups of evaluated CGM profiles were used to define the Q-Score limits between categories of very good, good, satisfactory, fair, and poor glycaemic control. In addition, we conducted a proof-of-principle study to show that patients with diabetes could be stratified for treatment based on the Q-Score using the CGM profiles of 1,562 historical subjects. The category of good glycaemic control included the majority of subjects treated with diet, half of those treated with OHA, only a quarter of those treated with OHA + insulin, and less than 20% of those treated with insulin alone. These findings are in accordance with other studies that demonstrate that blood glucose profiles are worsened with increasing therapy complexity (from diet alone to insulin) [25-27,37]. Thus, the categorical Q-Score allows the identification of subjects with poor metabolic control who require therapy optimisation. We intend to verify the Q-Score in a larger-scale study that includes CGM profiles derived from hospitalised subjects and those with diabetes and coexistent chronic illness.

Patient-tailored diabetes therapy represents the state-of the art in diabetes care and management [38,39]. Therefore, in addition to providing a general assessment of glycaemia, we demonstrate a method for identification of Q-Score parameters that require therapeutic attention and would provide a basis for personalised diabetes therapy. Profiles of people with diabetes categorised as very good or good were used to set the limit for the improvement potential of all Q-Score parameters. This method reveals the parameters that require therapeutic action; for example, the adjustment of the insulin therapy in the case of hypoglycaemia.

## Conclusions

We developed a metric for automated, objective evaluations of CGM profiles called the Q-Score. The Q-Score included the essential factors describing a glucose profile and therefore provides global information on glucose profiles, summarised in one value. The Q-Score can be categorised, and is suitable for screening profiles of individuals with insufficient metabolic control. In addition, it allows identification of factor(s) underlying the profiles that are mainly responsible for the quality of metabolic control in a given patient. Those parameters with improvement potential can be identified and addressed by therapeutic actions. Therefore, the Q-Score may efficiently contribute to designing strategies for patient-tailored diabetes care and management.

## Additional file

Additional file 1: Figure S1. Relationship between mean glucose and time that blood glucose remained above 8.9 mmol/L. The function indicates that, for a given mean glucose, the time spent in the hyperglycaemic range can vary for several hours. Even at a mean of 7 mmol/L the range is from 0 to 6 hours. At 9 mmol/L it is about 8–14 hours. Data are from 1562 profiles. **Figure S2.** Components of the Q-Score. Schematic illustration of the Q-Score components: MBG (mean glucose), MODD (mean of daily differences), t_hyper (_time in hyperglycaemia), t_hypo_ (time in hypoglycaemia), Range, (min-max-difference on one day). **Figure S3.** Representative examples of CGMs in different Q-Score categories. The Q-Score is given for each example in the upper right corner. The green region indicates the target range for glucose (3.9–8.9 mmol/L). **Figure S4.** Improvement potential increases for CGM profiles categorised from very good to poor. Number of parameters with improvement potential in five Q-Score categories. The highest number of parameters with improvement potential was found in CGM profiles categorised as poor. Data are from 1562 profiles. **Table S1.** Association of the Q-Score with CGM quality parameters. **Table S2.** Limits for the improvement potential categories given for all parameters of the Q-Score.

## Abbreviations

AUC_G_: Area under the curve for glucose
BU: Bread units
CHO: Carbohydrate intake
CGM: Continuous glucose monitoring
Q-Score: Quality evaluation score
MBG: Mean blood glucose
MAGE: Mean amplitude of glycaemic excursions
CONGA: Continuous overall net glycaemic action
MODD: Mean of daily differences
LBGI: Low glucose index
HBGI: High glucose index
GRADE: Glycaemic risk assessment diabetes equation
OHA: Oral hypoglycaemic agent
t_G_ time: Outside glucose target range
T1DM: Type 1 diabetes mellitus
T2DM: Type 2 diabetes mellitus
