# Supplementary material for: Q-Score: development of a new metric for continuous glucose monitoring that enables stratification of antihyperglycaemic therapies
Source: BMC Endocr Disord. 2015 May 1;15:22. doi: 10.1186/s12902-015-0019-0 (PMC4447008; doi:10.1186/s12902-015-0019-0)
Supplement: Additional file 1: Figure S1. — Relationship between mean glucose and time that blood glucose remained above 8.9 mmol/L. The function indicates that, for a given mean glucose, the time spent in the hyperglycaemic range can vary for several hours. Even at a mean of 7 mmol/L the range is from 0 to 6 hours. At 9 mmol/L it is about 8–14 hours. Data are from 1562 profiles. Figure S2. Components of the Q-Score. Schematic illustration of the Q-Score components: MBG (mean glucose), MODD (mean of daily differences), thyper (time in hyperglycaemia), thypo (time in hypoglycaemia), Range, (min-max-difference on one day). Figure S3. Representative examples of CGMs in different Q-Score categories. The Q-Score is given for each example in the upper right corner. The green region indicates the target range for glucose (3.9–8.9 mmol/L). Figure S4. Improvement potential increases for CGM profiles categorised from very good to poor. Number of parameters with improvement potential in five Q-Score categories. The highest number of parameters with improvement potential was found in CGM profiles categorised as poor. Data are from 1562 profiles. Table S1. Association of the Q-Score with CGM quality parameters. Table S2. Limits for the improvement potential categories given for all parameters of the Q-Score. [file 12902_2015_19_MOESM1_ESM.docx]

**Additional files**


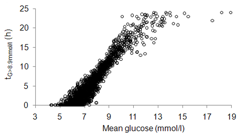


**Figure S1**

**Relationship between mean glucose and time that blood glucose remained above 8.9 mmol/L.** The function indicates that, for a given mean glucose, the time spent in the hyperglycaemic range can vary for several hours. Even at a mean of 7 mmol/L the range is from 0 to 6 hours. At 9 mmol/L it is about 8–14 hours. Data are from 1,562 profiles.

**
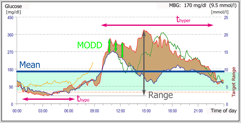
**

**Figure S2**

**Components of the Q-Score**

Schematic illustration of the Q-Score components: MBG (mean glucose), MODD (mean of daily differences), t_hyper (_time in hyperglycaemia), t_hypo_ (time in hypoglycaemia), Range, (min-max-difference on one day).

**
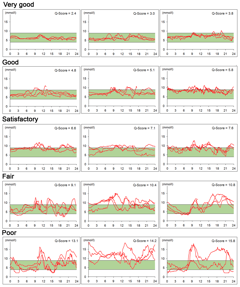
**

**Figure S3**

**Representative examples of CGMs in different Q-Score categories.** The Q-Score is given for each example in the upper right corner. The green region indicates the target range for glucose (3.9–8.9 mmol/L).


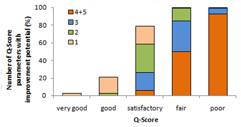


**Figure S4. Improvement potential increases for CGM profiles categorised from very good to poor**

Number of parameters with improvement potential in five Q-Score categories. The highest number of parameters with improvement potential was found in CGM profiles categorised as poor. Data are from 1562 profiles.

**Additional tables**

**Table S1**

**Association of the Q-Score with CGM quality parameters.**

|  | Q-Score categories | | | | |
| --- | --- | --- | --- | --- | --- |
| Parameter | Very good  (n=171)  11% | Good  (n=355)  23% | Satisfactory  (n=414)  27% | Fair  (n=383)  24% | Poor  (n=239)  15% |
| Q-Score | 3.3 ± 0.5 | 5.0 ± 0.6 | 7.2 ± 0.7 | 10.0 ± 1.0 | 14.3 ± 1.9 |
| HbA1c (%) | 6.4 ± 0.7 | 6.6 ± 0.6 | 6.9 ± 0.7 | 7.3 ± 0.9 | 7.8 ± 1.2 |
| HbA1c (mmol/mol) | 46.5 ± 7.6 | 48.6 ± 6.6 | 51.9 ± 7.7 | 56.3 ± 9.8 | 61.8 ± 13.1 |
| MBG (mmol/l) | 6.3 ± 0.5 | 6.8 ± 0.7 | 7.4 ± 0.8 | 8.3 ± 1.2 | 10.1 ± 2.3 |
| Range (mmol/l) | 3.8 ± 0.8 | 5.4 ± 0.9 | 7.1 ± 1.3 | 9.0 ± 1.8 | 11.6 ± 2.6 |
| SD (mmol/l) | 0.8 ± 0.2 | 1.2 ± 0.2 | 1.6 ± 0.3 | 2.1 ± 0.5 | 2.9 ± 0.8 |
| MAGE (mmol/l) | 2.1 ± 0.5 | 3.1 ± 0.8 | 4.3 ± 1.0 | 5.7 ± 1.5 | 7.5 ± 2.4 |
| t_G<3.9mmol/l_ (min) | 0 (0-0) | 0 (0-22) | 7 (0-55) | 10 (0-68) | 8 (0-92) |
| AUC_G<3.9mmol/l_  (mmol•h/l) | 0.0 (0-0) | 0.0 (0-0.12) | 0.03 (0-0.44) | 0.06 (0-0.7) | 0.08 (0-1.1) |
| LBGI | 0.2 (0.1-0.4) | 0.2 (0.0-0.5) | 0.2 (0.1-0.6) | 0.2 (0.0-0.7) | 0.2 (0.0-0.9) |
| t_G>8.9mmol/l_ (h) | 0.2 (0.0-0.6) | 1.6 (0.9-2.7) | 4.7 (3.2-6.6) | 9.0 (6-12) | 14.4 (10-19) |
| AUC_G>8.9mmol/l_ (mmol•h/l) | 0.0 (0.0-0.2) | 1.2 (0.5-2.4) | 5.9 (3.6-8.4) | 16.8 (11-24) | 46.8 (29-68) |
| HBGI | 0.2 (0.1-0.3) | 0.6 (0.4-0.9) | 1.4 (1.0-1.8) | 2.7 (1.8-3.6) | 5.7 (3.7-8.0) |
| GRADE | 2.1 ± 0.9 | 3.4 ± 1.1 | 5.0 ± 1.4 | 7.4 ± 2.0 | 12.1 ± 4.2 |
| MODD (mmol/l) | 0.8 ± 0.2 | 1.2 ± 0.3 | 1.6 ± 0.4 | 2.2 ± 0.5 | 3.2 ± 1.0 |

Quality parameters generally applied to describe CGM profiles were calculated for subjects (N=1562) in different Q-Score categories. Data represent the mean ± SD or median (interquartile range) for each category; p<0.001 for all variables.

AUC_G_, area under the curve for glucose; GRADE, glycaemic risk assessment diabetes equation; HbA1c, glycosylated haemoglobin; HBGI, high blood glucose index; LBGI, low blood glucose index; MAGE, mean amplitude of glycaemic excursions; MBG, mean blood glucose; MODD, mean of daily differences; SD, standard deviation; t_G_, glucose time outside target range

**Table S2**

**Limits for the improvement potential categories given for all parameters of the Q-Score.**

|  | Improvement potential | | | |
| --- | --- | --- | --- | --- |
| Q-Score Parameter | No | Low | Moderate | High |
| Mean (mmol/l) | < 8.0 | 8.0 - 10.0 | 10.0 - 12.0 | ≥ 12 |
| Range (mmol/l) | < 7.0 | 7.0 - 10.0 | 10.0 - 13.0 | ≥ 13 |
| t_G>8.9 mmol/l_ (h) | < 5.0 | 5.0 - 9.0 | 9.0 - 13.0 | ≥ 13 |
| t_G<3.9 mmol/l_ (h) | < 1.0 | 1.0 - 1.5 | 1.5 - 2.0 | ≥ 2.0 |
| MODD (mmol/l) | < 1.7 | 1.7 - 2.4 | 2.4 - 3.1 | ≥ 3.1 |

MODD, mean of daily differences; t_G_, glucose time outside target range
